# SHIP1 Activator AQX-1125 Regulates Osteogenesis and Osteoclastogenesis Through PI3K/Akt and NF-κb Signaling

**DOI:** 10.3389/fcell.2022.826023

**Published:** 2022-04-04

**Authors:** Xudong Xie, Liangcong Hu, Bobin Mi, Adriana C. Panayi, Hang Xue, Yiqiang Hu, Guodong Liu, Lang Chen, Chenchen Yan, Kangkang Zha, Ze Lin, Wu Zhou, Fei Gao, Guohui Liu

**Affiliations:** ^1^ Department of Orthopedics, Union Hospital, Tongji Medical College, Huazhong University of Science and Technology, Wuhan, China; ^2^ Hubei Province Key Laboratory of Oral and Maxillofacial Development and Regeneration, Wuhan, China; ^3^ Division of Plastic Surgery, Brigham and Women’s Hospital, Harvard Medical School, Boston, MA, United States; ^4^ Medical Center of Trauma and War Injuries, Daping Hospital, Army Medical University, Chongqing, China

**Keywords:** AQX-1125, SHIP1, bone loss, osteoblast, osteoclast

## Abstract

With the worldwide aging population, the prevalence of osteoporosis is on the rise, particularly the number of postmenopausal women with the condition. However, the various adverse side effects associated with the currently available treatment options underscore the need to develop novel therapies. In this study, we investigated the use of AQX-1125, a novel clinical-stage activator of inositol phosphatase-1 (SHIP1), in ovariectomized (OVX) mice, identifying a protective role. We then found that the effect was likely due to increased osteogenesis and mineralization and decreased osteoclastogenesis caused by AQX-1125 in a time- and dose-dependent manner. The effect against OVX-induced bone loss was identified to be SHIP1-dependent as pretreatment of BMSCs and BMMs with SHIP1 RNAi could greatly diminish the osteoprotective effects. Furthermore, SHIP1 RNAi administration *in vivo* induced significant bone loss and decreased bone mass. Mechanistically, AQX-1125 upregulated the expression level and activity of SHIP1, followed upregulating the phosphorylation levels of PI3K and Akt to promote osteoblast-related gene expressions, including Alp, cbfa1, Col1a1, and osteocalcin (OCN). NF-κB signaling was also inhibited through suppression of the phosphorylation of IκBα and P65 induced by RANKL, resulting in diminished osteoclastogenesis. Taken together, our results demonstrate that AQX-1125 may be a promising candidate for preventing and treating bone loss.

## Introduction

Bone homeostasis requires osteoclast-mediated removal of old or damaged bones and osteoblast-mediated formation of new bones. An imbalanced process, either due to excessive bone resorption and/or decreased bone formation, can lead to bone quality deterioration (Recker et al., 2004; Tranquilli Leali et al., 2009) and result in various bone conditions, including osteoporosis. Furthermore, the worldwide prevalence of osteoporosis is on the rise due to the aging population, which has also resulted in a growing number of postmenopausal women with osteoporosis (Looker et al., 2012). However, given the various severe side effects associated with currently available treatments, it is crucial to develop novel therapies to treat such conditions (Brumsen et al., 1997; Knopp-Sihota et al., 2013; Compston et al., 2019).

Increased bone formation, decreased bone resorption, or a combination of both effects may be a crucial therapeutic target for the treatment of osteoporosis. Osteoclasts, which are responsible for bone resorption, are tissue-specific multinucleated giant and cooperatively induced by the nuclear factor-κB ligand (RANKL) and the macrophage colony-stimulating factor (M-CSF) (Teitelbaum and Ross, 2003). The association of RANKL and its receptor RANKL recruits tumor necrosis factor receptor-associated factor 6 (TRAF6) and subsequent activation of downstream signaling molecules, including nuclear factor kappa-B (NF-κB), fos proto-oncogene (c-Fos), and mitogen-activated protein kinase (MAPK), leading to expression of osteoclast-related genes, such as the nuclear factor of activated T cells c1 (NFATc1) and tartrate-resistant acid phosphatase (TRAP) (Kim and Kim, 2016). Osteoblasts, the bone-forming cells, are derived from bone marrow mesenchymal stem cells (BMSCs) under the control of several transcription factors and signaling cascades. It has been shown that the Wnt/β-catenin pathway is involved in bone formation (Krishnan et al., 2006; Maupin et al., 2013), which promotes osteogenic differentiation of BMSCs through activation of Runt-related transcription factor 2 (Runx2) and inhibits peroxisome proliferator-activated receptor-γ (PPARγ) transcription to inhibit adipogenic differentiation of BMSCs (Bennett et al., 2005).

Studies have demonstrated that inositol phosphatase-1 (SHIP1) is strongly associated with the development of osteoporosis (Iyer et al., 2013). Specifically, SHIP^−/−^ mice exhibit severe osteoporosis, which is considered to be the result of excessive osteoclast activity (Takeshita et al., 2002). Mechanistically, src homology 2 (SH2) domain–containing inositol phosphatase-1 (SHIP1) can suppress TREM2- and DAP12-induced signaling *via* connection with DAP12 in an SH2 domain–dependent manner and restrict the recruitment of PI3K to DAP12 (Peng et al., 2010). Furthermore, hematopoietic stem cells (HSCs) from SHIP-deficient mice exhibit defective repopulating and self-renewal capacity upon transfer to SHIP-competent hosts (Desponts et al., 2006). In addition, lower alkaline phosphatase (Alp) activity, which is required for bone formation mediated by osteoblasts, has been noted in bone marrow–derived SHIP1^−/−^ osteoblasts, suggesting that osteoblast development and function might be directly impaired by SHIP1 deficiency (Hazen et al., 2009). Nonetheless, the aforementioned findings strongly indicate that SHIP1 is critical to maintain the balance between bone formation and bone resorption.

AQX-1125, also known as (1S,3S,4R)-4-[(3aS,4R,5S,7aS)-4-(aminomethyl)-7a–methyl-1-methylidene-octahydro-1H-inden-5-yl]-3-(hydroxymethyl)-4-methylcyclohexan-1-ol; acetic acid salt, is a novel, clinical-stage, low molecular weight activator of SHIP1, which has exhibited promising anti-inflammatory effects in a number of animal models of pulmonary inflammation (Stenton et al., 2013). Furthermore, oral AQX-1125 treatment of women with moderate to severe interstitial cystitis/bladder pain syndrome showed no serious adverse events, displaying a good tolerance profile (Nickel et al., 2016). As the effect of AQX-1125 on osteogenesis or/and osteoclastogenesis is yet to be established, in this study, we first carried out experiments *in vitro* to observe the molecule effect on osteogenesis or/and osteoclastogenesis, followed by *in vivo* demonstration of its protective functions in OVX-induced bone loss model.

## Materials and Methods

### Reagents

AQX-1125 was purchased from MedChemExpress (MCE). The primary antibodies of GAPDH, PI3K, p-PI3K, Akt, p-AKT, P65, p-P65, IκBα, p- IκBα, SHIP1, Runx2, Alp, NFATc1, and c-Fos were acquired from ABclonal (Wuhan, China). Phalloidin and 4, 6-diamidino-2-phenylindole (DAPI) were purchased from Solarbio (Beijing, China). RANKL (the receptor activator of the nuclear factor kappa-B ligand) and M-CSF (macrophage colony-stimulating factor) were obtained from R&D Systems (Minnesota, USA). Cell culturing plates were purchased from NEST (Jiangsu, China). Minimum Essential Medium Alpha (α-MEM), Dulbecco’s Modified Eagle Medium: F-12 (DMEM/F-12), fetal bovine serum (FBS), penicillin, streptomycin, and trypsin were purchased from Gibco (Grand Island, NY, United States). The TRAP staining kit was obtained from Solarbio (Beijing, China).

### Animal Model and Treatment

The ovariectomized mouse model was performed in specific pathogen-free (SPF) facilities, as previously described (Chen et al., 2017). All animal studies were performed according to protocols approved by the Laboratory Animal Center, Tongji Medical College, Huazhong University of Science and Technology, and were carried out as regulated by the Tongji Medical College Animal Care and Use Committee. Eight-week-old female C57BL/6 mice were randomly distributed into three groups (*n* = 3): the sham group (served as controls), model group (mice subjected to bilateral OVX and treated with vehicle), and treatment group (mice underwent bilateral OVX and treated with AQX-1125).

Briefly, mice were weighed and given general anesthesia with 1% pentobarbital by intraperitoneal injection and then subjected to bilateral OVX or a sham operation. Four weeks later, mice were treated with AQX-1125 (10 mg/kg) through intraperitoneal administration in the treatment group three times per week for 4 weeks. SHIP1-RNAi (5 nmol/20 g) was administrated through the tail vein to the mice in the treatment group twice a week for 4 weeks.

### Preparation and Culture of BMSCs

Human bone marrow specimens were collected from the iliac crests of healthy volunteer donors. All samples were attained with signed informed consent. BMSCs were isolated by density gradient centrifugation, and adherent cells were harvested. Cells were cultured in DMEM/F12 medium containing 10% FBS and 1% penicillin–streptomycin solution. The cells from the third passage were used in subsequent experiments.

### Preparation and Culture of Osteoclasts

Five-week-old male C57BL/6 mice were obtained from the Center of Experimental Animal, Tongji Medical College, Huazhong University of Science and Technology. Bone marrow–derived macrophages (BMMs) were prepared, as previously described (Xie et al., 2014). Briefly, bone marrow cells were obtained from mouse femurs and tibias. After elimination of red blood cells (RBCs) using RBC lysis buffer (Servicebio; Wuhan, China), the cells were incubated overnight in α-MEM medium (Gibco; Grand Island, NY, United States) supplemented with 10% FBS (Gibco) and 1% penicillin–streptomycin solution (Gibco). Nonadherent cells (stroma-free bone marrow cells) were incubated with 30 ng/ml M-CSF for 5 days to differentiate into osteoclast precursors (BMMs), which were then seeded into a 96-well culture plate at 1 × 10^4^ cells/0.2 ml/well and differentiated into mature osteoclasts with 30 ng/ml M-CSF and 50 ng/ml RANKL.

### Cell Counting Kit 8

For BMSCs, cells were seeded at a density of 5  ×  10^3^ cells per well in 96-well plates and cultured overnight. Then, cell viability was tested 3 days after AQX-1125 treatment. For the BMMs, approximately 1 × 10^4^ cells were seeded in 96-well plates and cultured overnight. Then, cells were incubated with various concentrations of AQX-1125 for 5 days in the presence of 30 ng/ml M-CSF and 50 ng/ml RANKL. The medium was replaced with serum-free medium containing the CCK-8 reagent and incubated for 2 h, followed by detection of absorbance at 450 nm.

### Osteoclastogenesis Assay *In Vitro*

Approximately 1 × 10^4^ BMMs were seeded into each well of a 96-well plate and cultured overnight. A differentiation medium (α-MEM medium, 10% fetal bovine serum, 1% penicillin–streptomycin solution, 30 ng/ml M-CSF, and 50 ng/ml RANKL) with or without AQX-1125 was used to induce osteoclast differentiation. After 5 days, TRAP staining was performed following the manufacturer’s instructions. TRAP-positive cells with more than three nuclei were regarded as osteoclasts. The osteoclastogenic ability was determined by analyzing the TRAP-positive area compared with the total area.

### Pit Formation Assay

Approximately 1 × 104 BMMs were seeded into each well of a 96-well plate and cultured overnight. Then, cells were incubated without AQX-1125 in the presence of 30 ng/ml M-CSF and 50 ng/ml RANKL and the medium was changed every 2 days. After 5 days, TRAP staining was performed to detect the osteoclast formation. Then, osteoclasts were digested with collagenase and seeded into a Corning® Osteo Assay Surface 96-well Plate, and cultured for 4 days with AQX-1125 in the presence of 30 ng/ml M-CSF and 50 ng/ml RANKL. The culture medium was replaced and the surface was washed with 0.5% sodium hypochlorite. The plate was washed twice with PBS and left to dry at room temperature for 3 h. Finally, the resorbing area was visualized using a digital microscope system and analyzed with the ImageJ software.

### Western Blotting

Cells (4 × 10^5^ cells per well) were seeded into six-well plates and cultured overnight. After treatment, total protein was extracted using a RIPA lysis buffer (50 mM Tris, 150 mM NaCl, 1% NP-40, and 0.5% sodium deoxycholate) with a proteinase inhibitor cocktail. Subsequently, 10 µg protein was subjected to 10% or 12.5% SDS-PAGE and electrophoretically transferred onto polyvinylidene fluoride (PVDF) membranes. After blocking with nonfat milk for 2 h, the PVDF membranes were incubated with specific antibodies at 4°C overnight. Next, the membranes were washed three times, each time for 10 min with TBS-T, and incubated with anti-rabbit secondary antibodies for 1 h at room temperature. After washing thrice with TBS-T, the membranes were incubated in a chemiluminescent substrate and visualized using the Bio-Rad Image Capture system.

### Real-Time PCR

Total RNA was extracted using TRIzol (Invitrogen), according to standard protocols. The concentration and purity of RNA were measured at an optical density of 260 nm/280 nm. One microgram of total RNA was used to synthesize cDNA (Vazyme; Nanjing, China). Equal quantities of cDNA were subjected to qRT-PCR using the 2×AceQ qPCR SYBR Green Master Mix (Vazyme; Nanjing, China), while GAPDH was used as the control. The cycle threshold (Ct) values were collected. The ΔΔCt method was used to calculate the relative mRNA levels of each target gene. The primers are listed in Table 1.

**TABLE 1:** Primer sequences.

Gene name	Primer sequence (5′ to 3′)
NFATc1-forward	5′-GGA​GCG​GAG​AAA​CTT​TGC​G-3′
NFATc1-reverse	5′-GTG​ACA​CTA​GGG​GAC​ACA​TAA​CT-3′
c-Fos	5′-CGG​GTT​TCA​ACG​CCG​ACT​A-3′
c-Fos	5′-TTG​GCA​CTA​GAG​ACG​GAC​AGA-3′
CTSK-forward	5′-CTC​GGC​GTT​TAA​TTT​GGG​AGA-3′
CTSK-reverse	5′-TCG​AGA​GGG​AGG​TAT​TCT​GAG​T-3′
ACP5-forward	5′-CAC​TCC​CAC​CCT​GAG​ATT​TGT-3′
ACP5-reverse	5′-AAG​TAG​TGC​AGC​CCG​GAG​TA-3′
Alp-forward	5′-ACC​ACC​ACG​AGA​GTG​AAC​CA-3′
Alp-reverse	5′-CGT​TGT​CTG​AGT​ACC​AGT​CCC-3′
Cbfa1-forward	5′-TGG​TTA​CTG​TCA​TGG​CGG​GTA-3′
Cbfa1-reverse	5′-TCT​CAG​ATC​GTT​GAA​CCT​TGC​TA-3′
Col1a1-forward	5′-GTG​CGA​TGA​CGT​GAT​CTG​TGA-3′
Col1a1-reverse	5′-CGG​TGG​TTT​CTT​GGT​CGG​T-3′
Osteocalcin-forward	5′-GGC​GCT​ACC​TGT​ATC​AAT​GG-3′
Osteocalcin-reverse	5′-GTG​GTC​AGC​CAA​CTC​GTC​A-3′
Mouse GAPDH-forward	5′-ACC​CAG​AAG​ACT​GTG​GAT​GG-3′
Mouse GAPDH-reverse	5′-CAC​ATT​GGG​GGT​AGG​AAC​AC-3′
Human GAPDH-forward	5′-ACA​ACT​TTG​GTA​TCG​TGG​AAG​G-3′
Human GAPDH-reverse	5′-GCC​ATC​ACG​CCA​CAG​TTT​C-3′
Mouse SHIP1-forward	5′-GCC​CCT​GCA​TGG​GAA​ATC​AA-3′
Mouse SHIP1-reverse	5′-TGG​GTA​GCT​GGT​CAT​AAC​TCC-3′
Human SHIP1-forward	5′-GCG​TGC​TGT​ATC​GGA​ATT​GC-3′
Human SHIP1-reverse	5′-TGG​TGA​AGA​ACC​TCA​TGG​AGA​C-3′

### Immunofluorescence Staining

Approximately 1 × 10^4^ BMMs were seeded into each well of a 96-well plate and cultured overnight. For the actin ring formation assay, α-MEM medium supplemented with 10% FBS, 1% penicillin–streptomycin solution, 30 ng/ml M-CSF, and AQX-1125 (0, 50, 100, 200 nM) in the presence/absence of 50 ng/ml RANKL was used to induce osteoclast differentiation for 5 days. The cells were fixed in 4% paraformaldehyde (PFA) for 20 min at room temperature. Then, the cells were washed at least four times with PBS and stained with phalloidin (proteintech; Wuhan, China) for 1 h, followed by DAPI staining for 5 min. Finally, the cells were observed using a digital microscope system (IX81; Olympus, Japan).

For immunofluorescence staining of OCN, the femurs were collected from mice and fixed in 4% PFA for 4 days. The samples were subsequently decalcified for 2 weeks using 10% tetracycline-EDTA (Servicebio, Wuhan, China), and 4-μm-thick paraffin-embedded sections were prepared. Subsequently, the slices were dewaxed in xylene for 20 min and rehydrated with a graded series of alcohol for 5 min, followed by antigen recovery in the 10 mM citrate buffer. Endogenous peroxidase was quenched using 3% hydrogen peroxide for 15 min, and then, sections were blocked with 10% unimmunized donkey serum for 30 min after washing three times with PBS. After that, the slices were incubated with the OCN rabbit polyclonal antibody (1:100; ABcolonal, China) at 4°C overnight. The slices were washed three times with PBS and incubated with the horseradish peroxidase-conjugated secondary antibody (1:200; proteintech) at room temperature for 1 h, followed by DAPI staining for 5 min.

### SHIP1 Small Interfering RNA Transfection

BMMs and BMSCs were incubated with SHIP1 small interfering RNA (siRNA; GENE; Shanghai, China) or nontarget control (NTC) siRNA (GENE; Shanghai, China) using the transfection reagents for 48 h, according to the manufacturer’s instructions. We verified the SHIP1 knockdown efficiency using qRT-PCR and Western blotting, followed by testing the differentiation capacity of BMSCs into osteoblasts and BMMs into osteoclasts. To enhance stability in serums and transfection efficiency, cholesterol, and methylation-modified siRNA, *in vivo* experiments were performed.

### Micro-CT Analysis

Micro-computed tomography (micro-CT; Bruker SkyScan 1176 scanner mCT system) was used to analyze the femur structure. Setting the analysis conditions to 37 kV and 121 mA, 300 section planes were scanned. For the morphometric analysis, we obtained the following trabecular parameters, bone mineral density (BMD), bone volume/tissue volume (BV/TV), trabecular bone surface area/total value (BS/TV) and trabecular number (Tb. N).

### Hematoxylin and Eosin Staining and TRAP Staining

The femurs were collected from mice and fixed in 4% PFA for 4 days. The samples were subsequently decalcified for 2 weeks using 10% tetracycline-EDTA (Servicebio), and 4-μm-thick paraffin-embedded sections were prepared for H&E staining and TRAP staining for further analysis. H&E and TRAP staining were carried out according to the kit instructions (Solarbio, Beijing, China). The images were obtained using a microscope, and histological analyses were performed by ImageJ software.

### Alkaline Phosphatase and Alizarin Red S Staining

Osteogenic induction was performed by culturing cells in an osteogenic differentiation medium (Cyagen Biosciences) containing 10% (v/v) FBS, 1% (v/v) penicillin–streptomycin, 2 mM l-glutamine, 50 μM ascorbate, 10 mM β-glycerophosphate, and 100 nM dexamethasone. The culture medium was changed every 2–3 days. The BMSCs were induced in the osteogenic differentiation medium for 14 days. Cells were washed thrice with PBS, and then fixed with 4% PFA for 15 min. Finally, cells were stained with an Alkaline Phosphatase Assay Kit (Alp; Beyotime, Shanghai, China, #C3206), according to the manufacturer’s instructions.

Alizarin Red S staining was performed after 21 days of induction. After fixing with 4% PFA, each well was treated with Alizarin Red S solution (Cyagen Biosciences) and incubated in the dark for 30 min. Then, the images were obtained by microscopy. Finally, quantification of calcium deposition was performed by elution of AR-S with 10% (W/V) cetylpyridinium chloride in 10 mM sodium phosphate (PH 7.0) for 1 h at room temperature, and absorbance of the eluted dye was measured at 570 nm.

### Statistical Analysis

All experiments were performed at least three times and are presented as the mean ± standard deviation (SD). One-way analysis of variance for three groups and Student’s t-test for two groups were performed using GraphPad Prism 5.0 (GraphPad Software). A value of *p* < 0.05 was considered statistically significant.

## Results

### AQX-1125 Alleviates OVX-Induced Bone Loss *In Vivo*

**FIGURE 1:**
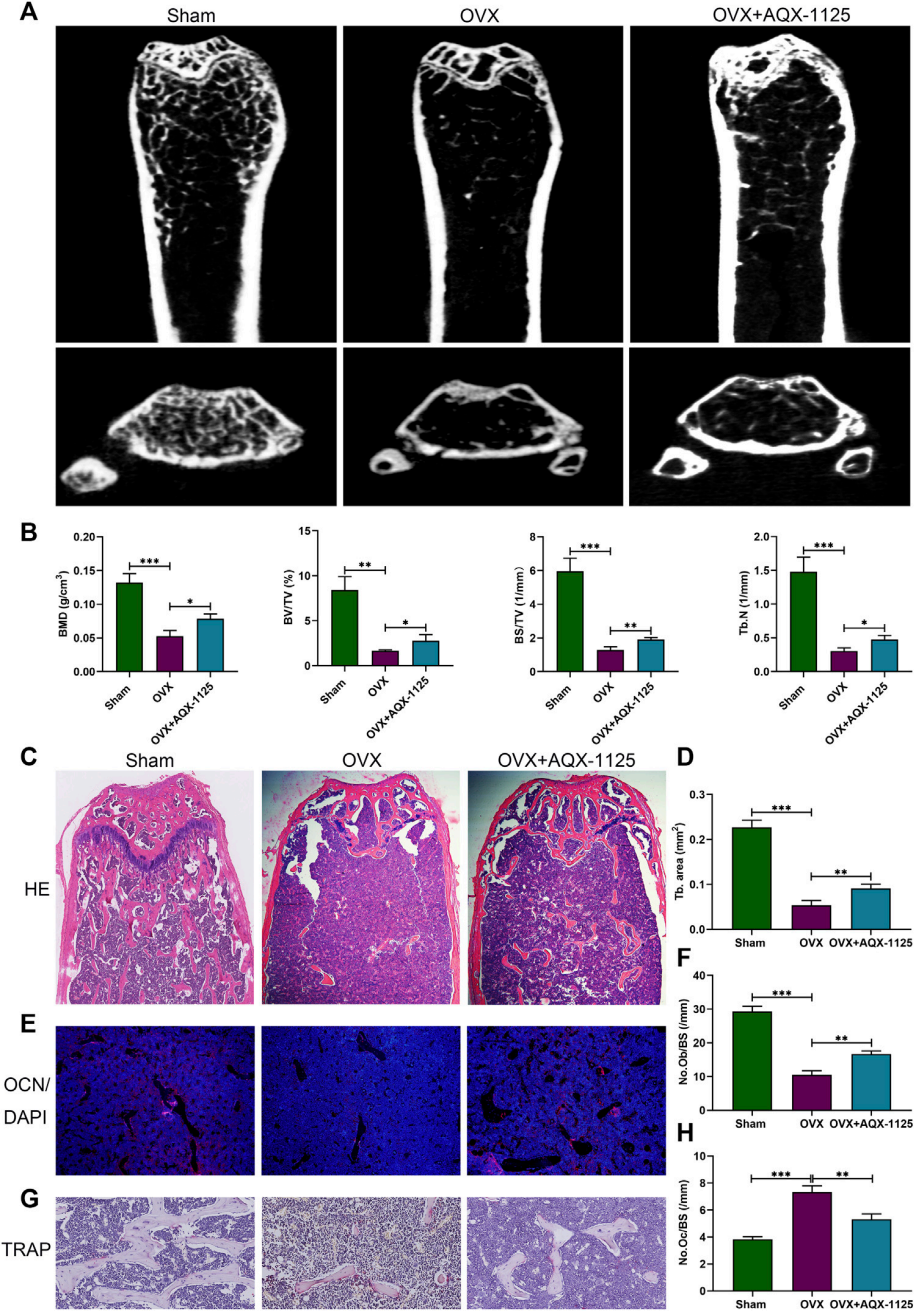
AQX-1125 alleviates OVX-induced bone loss *in vivo*. **(A)** Micro-CT analysis of the distal femurs from three groups (*n* = 3): sham, OVX, and OVX + AQX-1125 (10 mg/kg/bw). **(B)** Quantitative analysis of BMD, BV/TV, BS/TV and Tb. N. **(C)** Representative images of HE staining of the distal femur sections. Scale bars = 200 μm. **(D)** Quantitative analysis of trabecular (Tb) bone area in **(C)**. **(E)** Representative immunofluorescence images of OCN in femurs from different groups. Blue: DAPI; red: OCN. Scale bars = 100 μm. **(F)** Quantification of the osteoblast number in **(E)**. **(G)** Representative images of femurs stained with TRAP. Scale bars = 50 μm. **(H)** Histomorphometric analysis of the osteoclast number in **(G)**. Data are presented as means ± SD from three independent experiments (*p < 0.05; **p < 0.01; and ***p < 0.001).

To investigate whether AQX-1125 has protective effects in osteoporosis, we performed an ovariectomized mouse model mimicking postmenopausal osteoporosis. We observed extensive bone loss in the OVX groups using micro-CT. Intraperitoneal injection of AQX-1125 (10 mg/kg) for 4 weeks was shown to reduce the OVX-induced bone loss in the distal femur (Figure 1A). BMD, BV/TV, BS/TV and Tb.N were measured, and an increase in BMD, BV/TV, BS/TV, and Tb.N in the OVX + AQX-1125 group was observed compared to the OVX group (Figure 1B), as confirmed by H&E staining (Figures 1C,D). To investigate whether AQX-1125’s bone protective effects occurred through enhanced osteoblast differentiation and/or decreased osteoclast formation and activity, we performed immunofluorescence analysis of the levels of the OCN expression and TRAP staining in the femurs. As seen in Figures 1E,F, the OCN expression level was downregulated in OVX mice, while reduced level of the OCN expression was partly restored upon AQX-1125 stimulation. In addition, we also found that AQX-1125 administration could reduce the number of TRAP-positive cells in the femurs of ovariectomized mice (Figures 1G,H). Together, these results demonstrated that AQX-1125 could effectively rescue OVX-induced bone loss potentially *via* regulation of osteogenesis and osteoclastogenesis.

### AQX-1125 Facilitates Osteoblast Differentiation

**FIGURE 2:**
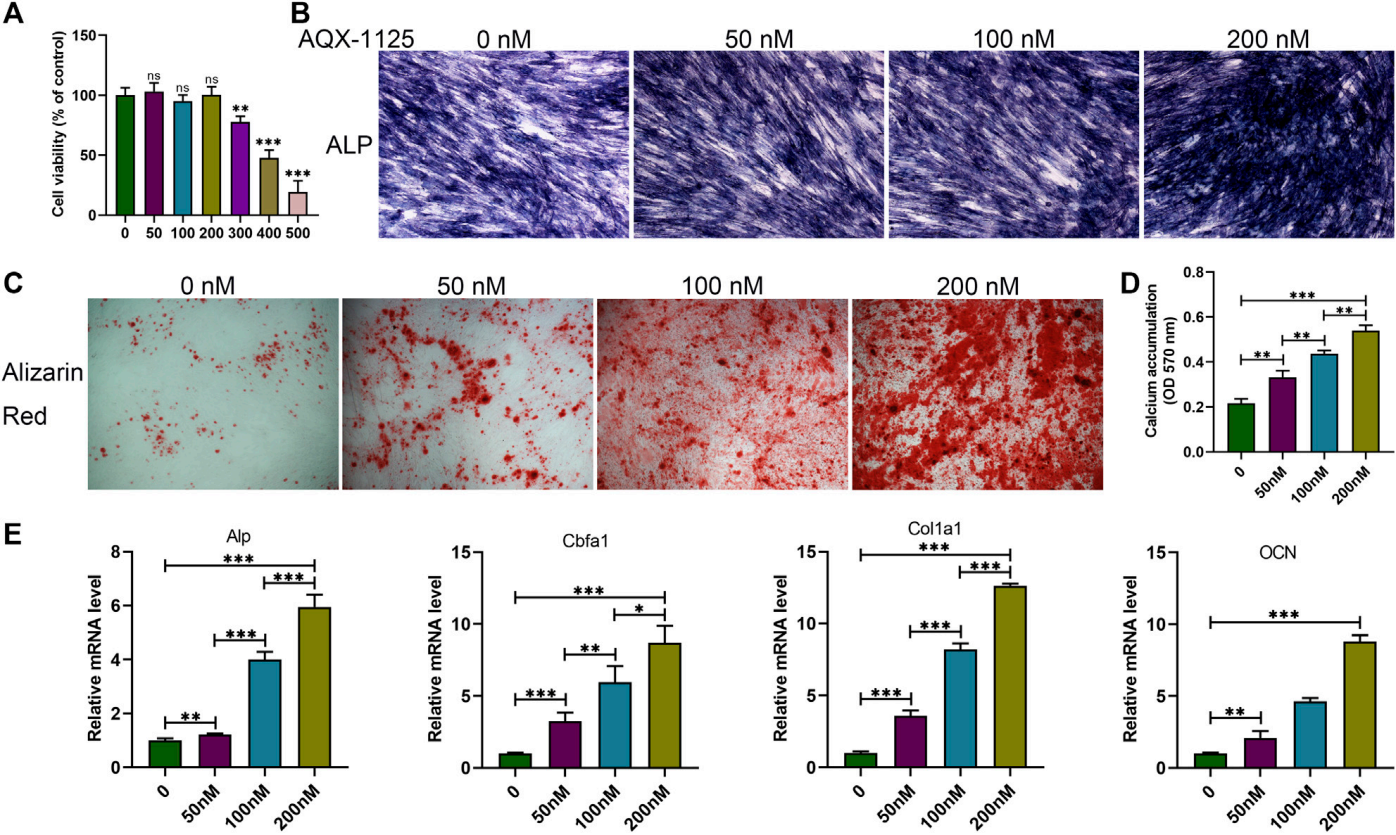
AQX-1125 facilitates osteoblast differentiation. **(A)** Effect of AQX-1125 on BMSCs viability. **(B,C)** BMSCs were cultured in osteogenic differentiation medium with various concentrations of AQX-1125, as indicated for 14 days (B, Alp staining) or 21 days (C, alizarin red staining. scale bars = 100 μm). **(D)** Matrix mineralization was assessed by quantitative analysis of alizarin red S staining in **(C)**. **(E)** Relative mRNA expression level of Alp, Cbfa1, Col1a1, and OCN in BMSCs treated with AQX-1125, as indicated for 24 h. Data are presented as means ± SD from three independent experiments (*p < 0.05; **p < 0.01; and ***p < 0.001).

We first investigated the influence of AQX-1125 on osteoblast formation *in vitro*. Before the study, a CCK-8 analysis was performed to determine the appropriate assay concentration of AQX-11125. As seen in Figure 2A, no toxic effects were observed after incubation with an AQX-1125 concentration lower than 200 nM. Alp and alizarin red S staining were performed to detect osteoblast differentiation and mineralization. Alp- and alizarin red S–positive cells increased in the presence of AQX-1125 in a dose-dependent manner, which demonstrated that AQX-1125 could enhance osteoblast differentiation and mineralization (Figures 2B–D). Consistently, the osteogenic markers, including Alp, Cbfa1, Col1a1, and OCN genes were also markedly upregulated after induction with AQX-1125 (Figure 2E). These data showed that BMSCs after exposure to AQX-1125 were in a state of enhanced osteogenesis.

### AQX-1125 Inhibits RANKL-Induced Osteoclast Differentiation *In Vitro*

**FIGURE 3:**
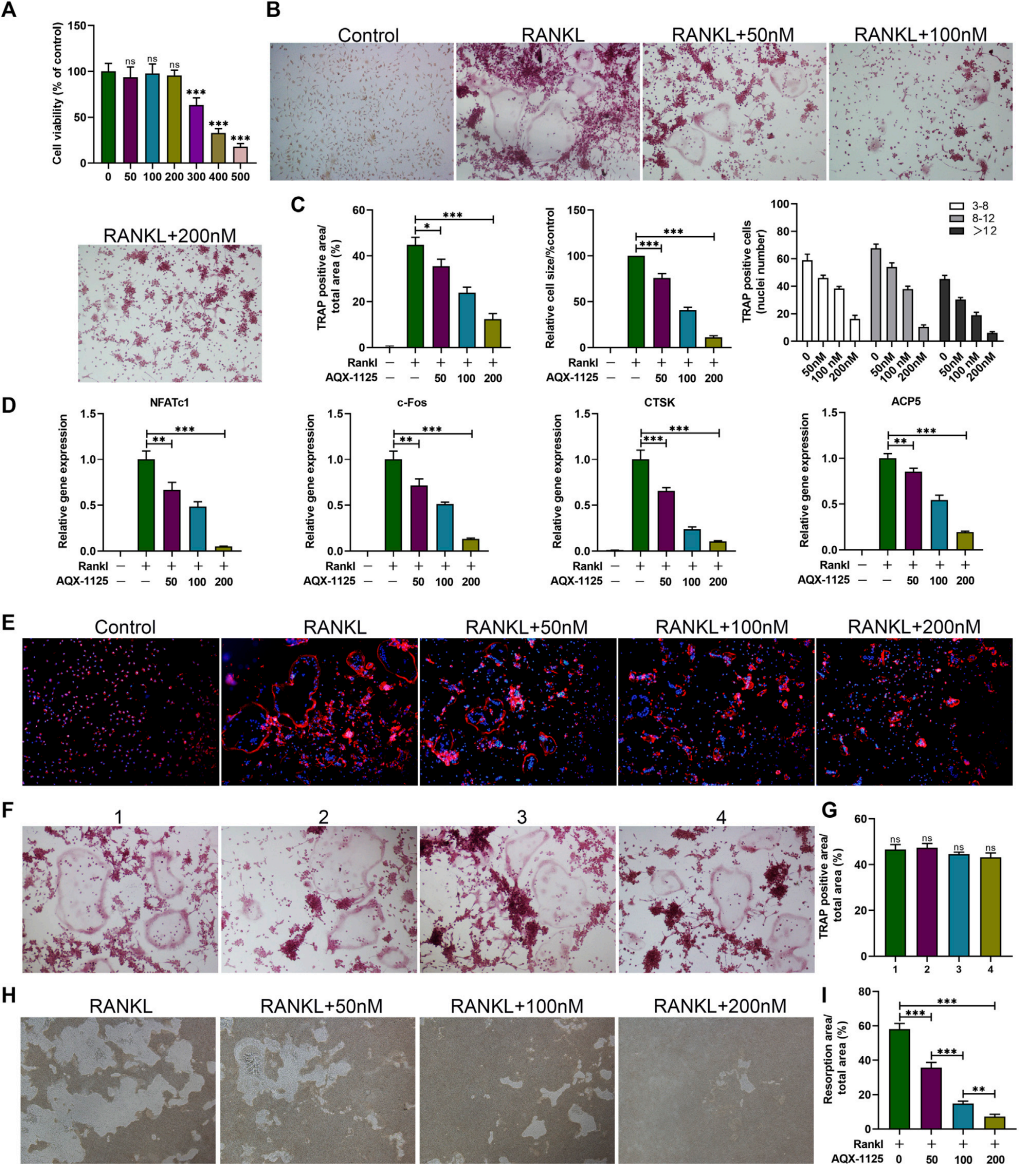
AQX-1125 inhibits RANKL-induced osteoclast differentiation *in vitro*. **(A)** Effect of AQX-1125 on BMMs viability. **(B)** BMMs were cultured in α-MEM medium with 30 ng/ml M-CSF and 50 ng/ml RANKL in the presence of various concentrations of AQX-1125, as indicated for 5 days. TRAP-staining and quantification of TRAP-positive cells. Scale bars = 100 μm. **(C)** Quantitative analysis was performed to assess TRAP-positive cells/total area, relative TRAP-positive cell size, and the number of nuclei in TRAP-positive cells. **(D)** BMMs were cultured for 24 h in the presence of different concentrations of AQX-1125 (0, 50, 100, 200 nM). The mRNA expression levels of NFATc1, c-Fos, Ctsk, and ACP5 were detected with qRT-PCR. **(E)** F-Actin ring formation assays were carried out to detect the effect of AQX-1125 on the generation of mature osteoclasts. The actin rings were detected using phalloidin by fluorescence microscopy following treatment with various concentrations of AQX-1125. Scale bars = 100 μm. **(F)** TRAP-staining and quantitation of TRAP-positive cells. BMMs were cultured for 5 days to differentiate into mature osteoclasts in differentiation medium in the absence of AQX-1125. Scale bars = 100 μm. **(G)** Quantitative analysis was performed to test TRAP-positive cells/total area. **(H,I)** Pit formation assay of osteoclasts and quantification of the pits area. The images were captured with a microscope. Scale bars = 100 μm. Data are presented as means ± SD from three independent experiments (*p < 0.05; **p < 0.01; ***p < 0.001).

To explore the effect of AQX-1125 on osteoclast formation *in vitro*, bone marrow macrophages (BMMs), a standard *in vitro* osteoclast differentiation model, were used. We performed CCK-8 assay to explore the effect of AQX-1125 on BMMs viability, suggesting that cell viability was comparable to that of the vehicle-treated cultures at lower doses below 200 nM (Figure 3A). After RANKL induction for 5 days, we observed significantly increased numbers of TRAP-positive cells. BMMs were then treated with AQX-1125 at various concentrations (0, 50, 100, 200 nM) in the presence of 30 ng/ml M-CSF and 50 ng/ml RANKL and we found that addition of AQX-1125 sharply suppressed osteoclast differentiation in a dose-dependent manner, as demonstrated by TRAP staining (Figure 3B). Osteoclasts with smaller size and fewer than eight nuclei were observed upon treatment with 200 nM AQX-1125 (Figure 3C), which was consistent with the qRT-PCR results (Figure 3D). Then, to evaluate the osteoclast activity, we detected actin ring formation of osteoclasts, which is considered to be a key process during the formation of mature osteoclasts. After treatment with various concentrations of AQX-1125, the actin ring became smaller in a dose-dependent manner, showing that AQX-1125 could impede actin ring formation in mature osteoclasts (Figure 3E). Furthermore, to verify the direct effect of AQX-1125 on osteoclast activity, we cultured BMMs in differentiation medium for 5 days in the absence of AQX-1125 and then verified osteoclast differentiation by TRAP staining (Figures 3F,G), which suggested that BMMs differentiated into osteoclasts, and no difference was seen in the osteoclast number and size in the absence of AQX-1125. Then, mature osteoclasts were cultured for 4 additional days with AQX-1125, and the bone resorption pits were analyzed to evaluate AQX-1125’s effect on the resorption activity of mature osteoclasts. The results indicated that treatment with AQX-1125 decreased the bone resorption area significantly compared with vehicle treatment (Figures 3H,I).

### AQX-1125 Promotes Osteoblast Differentiation *via* the PI3K/Akt Signaling Pathway

**FIGURE 4:**
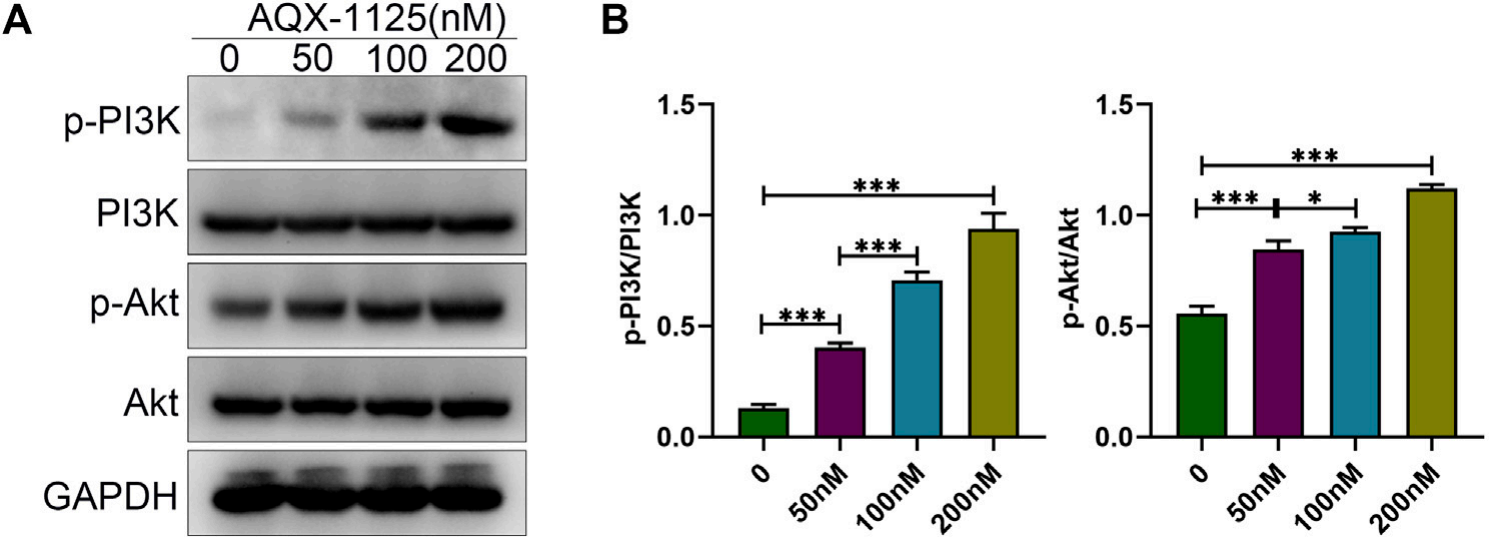
AQX-1125 promotes osteoblast differentiation *via* the PI3K/Akt signaling pathway. **(A)** Protein expression levels of PI3K, p-PI3K, Akt and p-Akt in BMSCs were detected by Western blotting after treatment with various concentration of AQX-1125 for 4 days. **(B)** Quantitative analysis of each immunoblot in **(A)**. Data are presented as means ± SD from three independent experiments (*p < 0.05; **p < 0.01; ***p < 0.001).

Studies have indicated that PI3K plays a central role in osteoblast differentiation and survival (Guntur and Rosen, 2011), and PI3K/Akt signaling activation by Wnt3a and heparin exhibits increased osteoblast differentiation (Ling et al., 2010). Therefore, we tested whether AQX-1125 increased osteoblast differentiation through activation of the PI3K/Akt signaling pathway. As shown in Figures 4A,B, Western blotting showed that treatment with AQX-1125 facilitated the phosphorylation of p-PI3K and p-Akt in BMSCs, which suggested that AQX-1125 significantly activated PI3K/Akt signaling pathway.

### AQX-1125 Suppresses the NF-κB Pathway during Osteoclastogenesis

**FIGURE 5:**
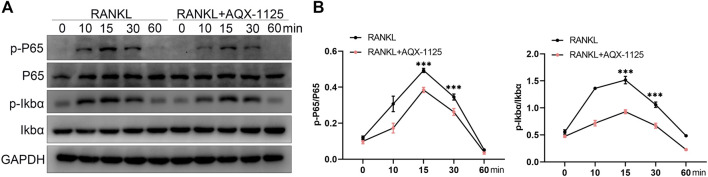
AQX-1125 suppresses the NF-κB pathway in osteoclastogenesis. **(A)** Western blotting was used to test the phosphorylation levels of IκBα and P65 in the presence of 50 ng/ml RANKL after pretreatment with AQX-1125 for the indicated time. **(B)** Quantitative analysis of the Western blotting results. Data are presented as means ± SD from three independent experiments (*p < 0.05; **p < 0.01; ***p < 0.001).

In the last decade, studies have established that NF-κB signaling is involved in RANKL–induced osteoclastogenesis, and it has been proven that inhibition of NF-κB signaling is an effective strategy to suppress osteoclast formation and bone resorption activity (Abu-Amer, 2013). Therefore, NF-κB signaling plays a predominant function in osteoclast differentiation and activity. To investigate whether AQX-1125 inhibited osteoclastogenesis *via* the NF-κB signaling mechanism, we performed Western blotting assays of the NF-κB signaling pathway. In BMMs, we observed that AQX-1125 suppressed the RANKL-induced phosphorylation of IκBα and P65 (Figures 5A,B). These data showed that the AQX-1125 treatment suppressed RANKL-mediated NF-κB signaling activation.

### Increased Osteogenesis and Decreased Osteoclastogenesis Induced by AQX-1125 are SHIP1-Dependent

**FIGURE 6:**
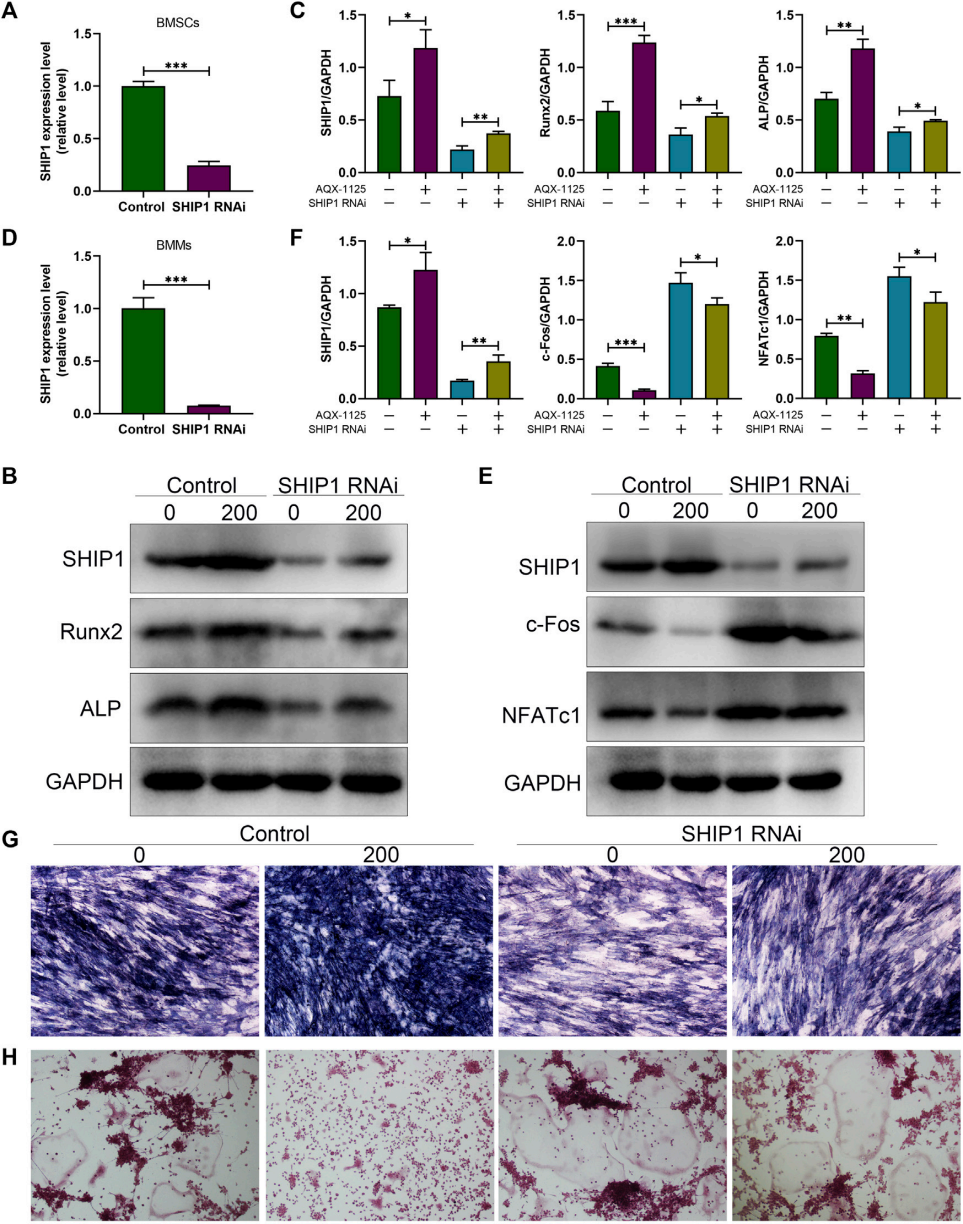
Increased osteogenesis and decreased osteoclastogenesis induced by AQX-1125 are SHIP1-dependent. **(A)** Gene expression level of SHIP1 in BMSCs after transfection with SHIP1 RNAi. **(B)** Protein expression levels of SHIP1, Runx2, and Alp in BMSCs with or without AQX-1125, were detected 4 days following transfection with SHIP1 RNAi. **(C)** Quantitative analysis of each immunoblot in **(B)**. **(D)** Gene expression level of SHIP1 in BMMs after transfection with SHIP1 RNAi. **(E)** BMMs were treated with or without AQX-1125 for 3 days in the presence of M-CSF and RANKL. The expression levels of SHIP1, NFATc1, and c-Fos was measured with Western blotting. **(F)** Quantitative analysis of each immunoblot in **(E)**. **(G)** BMSCs were transfected with SHIP1 RNAi for 48 h, and then, Alp staining was performed 14 days after osteogenic induction in the absence or presence of AQX-1125. Scale bars = 100 μm. **(H)** BMMs were incubated with SHIP1 RNAi for 48 h, and then TRAP staining was used to test the effect of SHIP1 RNAi on AQX-1125-mediated osteoclastogenesis after 5-days incubation with differentiation medium in the absence or presence of AQX-1125. TRAP staining was used to test the effect of SHIP1 RNAi on AQX-1125-mediated osteoclastogenesis. Scale bars = 100 μm. Data are presented as means ± SD from three independent experiments (*p < 0.05; **p < 0.01; and ***p < 0.001).

To demonstrate that AQX-1125 predominantly exerted its effects through activation of SHIP1, BMSCs and BMMs were first transfected with either control siRNA or siRNA against SHIP1. To verify the transfection efficiency, the expression of the SHIP1 expression levels in BMSCs and BMMs were detected. These results showed that SHIP1 RNAi (SHIP1 siRNA) significantly decreased the SHIP1 mRNA level in BMSCs (Figure 6A) and BMMs (Figure 6D). Subsequently, Western blotting showed that AQX-1125 significantly activated the SHIP1 expression, followed by an increase in the Runx2 and Alp expression level in BMSCs (Figures 6B,C) and a diminished NFATc1 and c-Fos expression level in BMMs (Figures 6E,F), while the effects could be markedly reversed after pretreatment with SHIP1 RNAi (Figures 6B,C,E,F). Furthermore, after pretreatment with SHIP1 RNAi, reduced Alp activity and staining and increased osteoclast formation were observed compared with the control group, which had received no treatment with SHIP1 RNAi (Figures 6G,H). The results highlighted that the protective role against OVX-induced bone loss was likely to be SHIP1-dependent.

### SHIP1 RNAi Administration Causes Bone Loss

**FIGURE 7:**
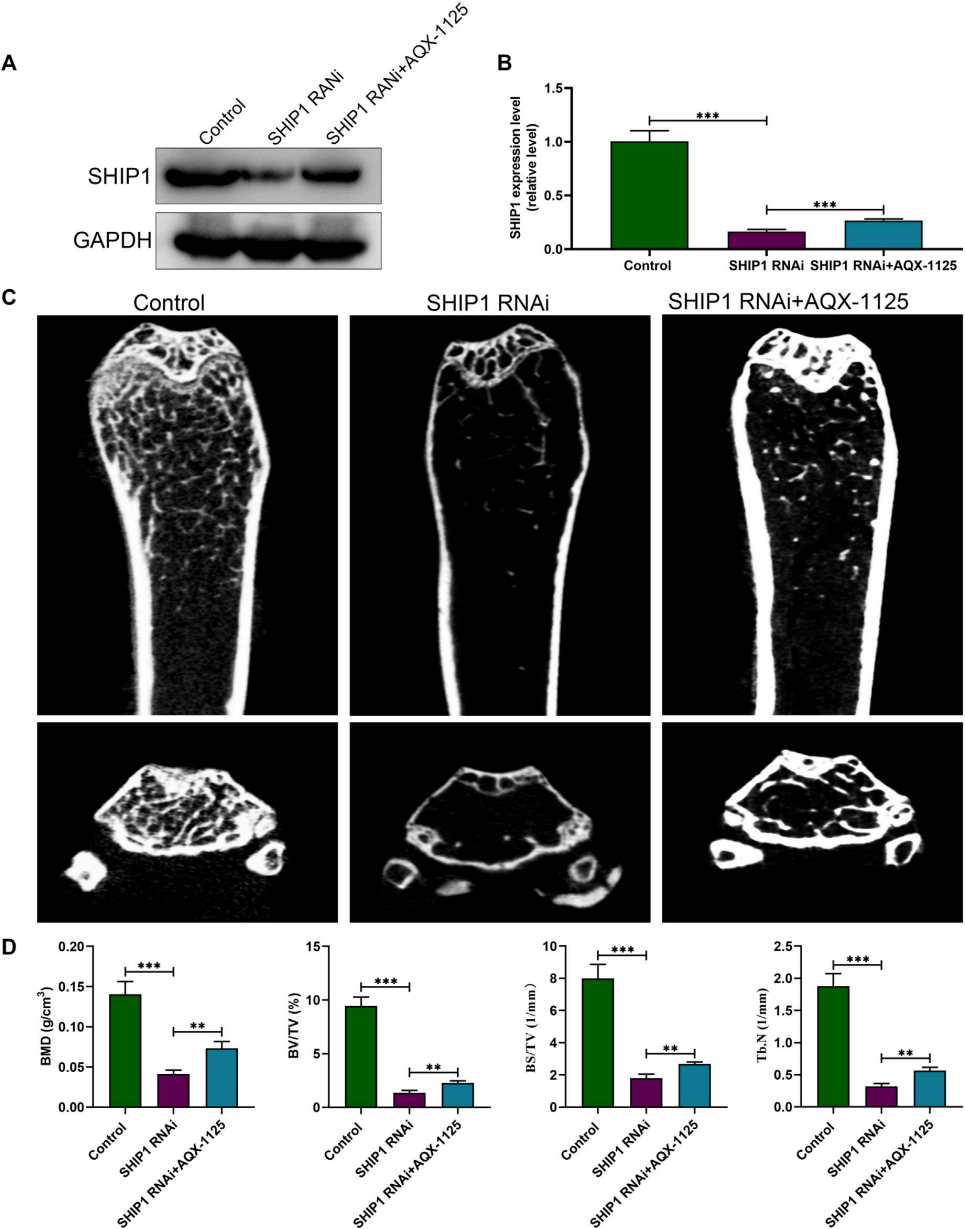
SHIP1 RNAi administration induces bone loss. **(A)** Protein expression level of SHIP1 in femurs after exposure to treatment. Eight-week-old male C57 mice were retreated with vehicle (control group), SHIP1 RNAi (5 nm/20 g), and SHIP1 RNAi (5 nm/20 g) +AQX-1125 (10 mg/kg) for 4 weeks. Total protein was isolated from femurs of each group, and Western blotting was performed. **(B)** Quantitative analysis of each immunoblot in **(A)**. **(C)** Micro CT analysis of the distal femurs from the control, SHIP1 RNAi, and SHIP1 RNAi + AQX-1125 group. **(D)** Calculation of the BMD, BV/TV, BS/TV and Tb.N. Data are presented as means ± SD from three independent experiments (*p < 0.05; **p < 0.01; ***p < 0.001).

After demonstrating that the protective effect of AQX-1125 against bone loss is SHIP1-dependent, we investigated whether SHIP1 RNAi administration could cause bone loss or deteriorate osteoporosis. Western blotting verified efficient knockdown of SHIP1 in femurs, following treatment with SHIP1 RNAi *via* the tail vein for 4 weeks, while AQX-1125 only partly reversed the effect of SHIP1 RNAi (Figures 7A,B). Furthermore, SHIP1 RNAi–treated mice displayed deteriorated trabecular bone microarchitecture in their femurs compared with the vehicle-treated group (Figure 7C). Morphometric analyses of trabecular parameters confirmed the decreased bone mass in BMD, BS/TV, BV/TV, and Tb.N in SHIP1 RNAi–treated mice, and the SHIP1 RNAi–induced deterioration of the trabecular microarchitecture was only partly reversed with AQX-1125 (Figure 7D). Together, AQX-1125, as an activator of SHIP1, is highly likely to exhibit the bone protective roles.

## Discussion

**FIGURE 8:**
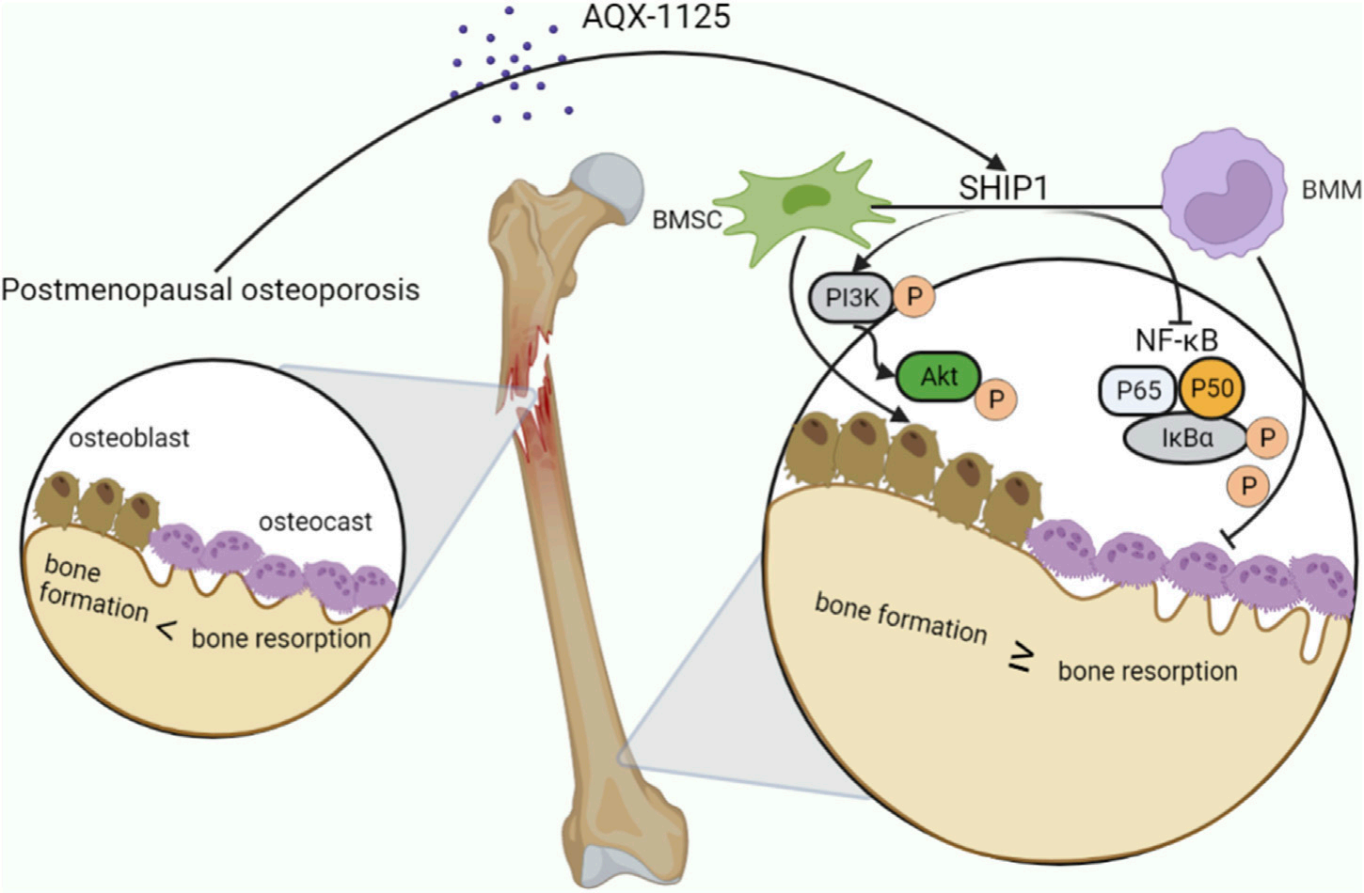
AQX-1125 positively regulates osteogenesis through PI3K/Akt signaling and inhibits osteoclastogenesis through NF-κb signaling *via* activation of SHIP1. Upon AQX-1125 stimulation, SHIP1 was activated in the BMSCs and BMMs. The activated SHIP1 facilitates the phosphorylation of PI3K and Akt in BMSCs, followed by acceleration of the differentiation of BMSCs into osteoblasts. In addition, activated SHIP1 inhibits the NF-κb signaling pathway, which reduces the phosphorylation of p65 and IkBα in BMMs, followed by suppression of the differentiation of BMMs into osteoclasts and the activity of osteoclasts. Overall, AQX-1125 alleviates OVX-induced bone loss.

In this study, our data demonstrate that AQX-1125 facilitates osteogenesis and inhibits osteoclastogenesis *in vitro* through SHIP1-dependent PI3K/Akt and NF-κB signaling pathway (Figure 8). Moreover, AQX-1125 rescued OVX-induced osteoporosis *in vivo*, while SHIP1 RNAi administration led to bone loss. Together, these findings indicate that AQX-1125 has promising potential as a novel drug for the treatment of osteoporosis.

Studies have demonstrated that AQX-1125 can exhibit a variety of biological effects, including inhibition of bleomycin-induced pulmonary fibrosis (Cross et al., 2017), relief of allergic and pulmonary inflammation *in vivo* (Stenton et al., 2013), and improvement of moderate to severe interstitial cystitis/bladder pain syndrome (Nickel et al., 2016), but no such evidence has been shown in bones. Therefore, we first explored the ability of AQX-1125 to treat osteoporosis. Here, AQX-1125 was used in OVX-induced osteoporosis model, and the results highlighted that the compound could sharply attenuate bone loss *in vivo*.

The balance between bone regeneration and resorption is dependent on the osteoblast and osteoclast population (Jakob et al., 2015). Disruption of the balance, for example, increased osteoclastogenesis and decreased osteogenesis under certain conditions, such as the estrogen deficiency, aging, and inflammation, results in bone loss or osteoporosis (Chen et al., 2016; Seo et al., 2016; Wang et al., 2016). Inhibition of osteoclastogenesis or/and promotion of osteogenesis can counteract bone loss under these conditions. To further investigate the mechanism of increased bone mass caused by AQX-1125, we performed *in vitro* experiments. We found that AQX-1125 could facilitate osteogenesis and mineralization in a dose-dependent manner. Additionally, treatment with AQX-1125 was associated with upregulation of osteoblast-related genes, including *Alp*, *cbfa1*, *Col1a1*, and OCN. The PI3K/Akt signaling pathway plays a crucial role in bone tissue not only by facilitating proliferation and differentiation but also by suppressing apoptosis in osteoblasts (Ling et al., 2010; Guntur and Rosen, 2011). We demonstrated that the compound might activate the PI3K/Akt signaling pathway to accelerate osteoblast differentiation and mineralization by Western blot assay, which exhibited that treatment with AQX-1125 significantly increased the phosphorylation of PI3K and subsequently increased the phosphorylation of Akt in a dose-dependent manner.

Additionally, we also observed that AQX-1125 suppressed RANKL-induced osteoclast differentiation and bone resorption in a dose-dependent manner *in vitro*. Osteoclast-related genes, such as *NFATc1*, *c-Fos*, *CTSK*, and *ACP5*, were downregulated following treatment with AQX-1125. From these, NFATc1 is considered to be a central regulator of osteoclast differentiation (Takayanagi et al., 2002). Mechanistically, we found that AQX-1125 could suppress the activation of RANKL-induced NF-κB signaling by inhibiting the phosphorylation of p65 and IκBα. These results show that AQX-1125-mediated inhibition of osteoclastogenesis might be involved in the NF-κB signaling pathway.

It is well established that PI3K/Akt and NF-κB are downstream signaling molecules in this process. Hence, we hypothesized the existence of an upstream molecule that regulates the effects upon stimulation with AQX-1125. Given that the compound is a novel, clinical-stage activator of SHIP1, we identified SHIP1 as the crucial upstream molecule. Moreover, prior research has shown that SHIP1 is associated with osteogenesis and osteoclastogenesis (Peng et al., 2010; Iyer et al., 2013). In our study, treatment with AQX-1125 markedly upregulated SHIP1 protein level, followed by an increase in the Alp and Runx2 expression levels and a concurrent decrease in the NFATc1 and c-Fos expression levels. Pretreatment with SHIP1 RNAi could reverse the effects. Accordingly, increased Alp activity and staining and decreased TRAP staining were observed with the use of AQX-1125, while SHIP1 RNAi significantly weakened the effects of stimulation of AQX-1125. Furthermore, SHIP1 RNAi administration *in vivo* resulted in bone loss. Our results highlight that the AQX-1125–induced protection against bone loss was SHIP1-dependent.

## Conclusion

Taken together, our results demonstrate that AQX-1125 promotes osteogenesis and suppresses osteoclastogenesis *in vitro* and attenuates OVX-induced bone loss *in vivo* through SHIP1-dependent PI3K/Akt and NF-κB signaling. Therefore, regulating the expression of SHIP1 with AQX-1125 could be a promising option for the treatment of osteoporosis in the future. However, our findings are based solely on the ovarectomized mouse model, with more clinical research warranted.

## Data Availability

The raw data supporting the conclusion of this article will be made available by the authors, without undue reservation.
